# Looking for the Key to Winning: Psychophysiological Predicting Factors in Healthy University Students

**DOI:** 10.3390/bs13120978

**Published:** 2023-11-27

**Authors:** Raquel Costa, Diana Abad-Tortosa, Adrian Alacreu-Crespo, Elena Saiz-Clar, Alicia Salvador, Miguel Ángel Serrano

**Affiliations:** 1 Departamento de Psicobiología, Universitat de València, 46010 Valencia, Spain; raquel.costa-ferrer@uv.es (R.C.); diana.abad@uv.es (D.A.-T.); alicia.salvador@uv.es (A.S.); 2 Departamento de Psicología y Sociología, Universidad de Zaragoza, 50009 Zaragoza, Spain; aalacreu@unizar.es; 3 Departamento de Metodología, Universitat Nacional a Distancia (UNED), 28040 Madrid, Spain; esaiz@bec.uned.es; 4 Laboratory of Social Cognitive Neuroscience and IDOCAL, Universitat de València, 46010 Valencia, Spain; 5 Spanish National Network for Research in Mental Health CIBERSAM, 28029 Madrid, Spain

**Keywords:** winner–loser, competitiveness, self-efficacy, motivation, anxiety, heart rate reactivity

## Abstract

Performance in competitive situations has been linked to various psychobiological factors such as personality traits (e.g., competitiveness), situational appraisal (e.g., motivation), and cardiovascular response (e.g., heart rate). However, it remains unclear whether these factors can predict competitive success. This paper aims to assess, through discriminant analysis, the predictive capacity of these psychobiological variables regarding the likelihood of winning, ultimately delineating a psychophysiological profile associated with success. Across three distinct studies, a total of 154 participants (66 men) engaged in a face-to-face laboratory competition. Prior to the competition, assessments of competitiveness traits, anxiety, self-efficacy, and motivation were conducted, and heart rate reactivity during the competition was measured. These variables collectively formed the basis for constructing the predictive model. The results of the initial study demonstrated that our model accurately classified 68.8% of the cases. Specifically, high levels of competitiveness, self-efficacy, motivation, and heart rate reactivity, coupled with low anxiety, were predictive of winning. These findings were subsequently replicated in two independent validation samples involving both men and women (studies 2 and 3), thereby reinforcing the robustness of the earlier results. In conclusion, our findings suggest that the psychological state preceding competition, along with cardiovascular reactivity, may serve as predictors for the probability of winning.

## 1. Introduction

Competitive situations are present in many areas of our lives, such as sports, academics, and work life. They constitute a prevalent means of attaining significant objectives, including but not limited to status, economic remuneration, and emotional well-being [1]. Due to its significance, the exploration of competition has a rich historical trajectory, and in recent years, there has been a growing recognition of the pivotal role played by individual differences when confronting competitive situations [2]. The coping competition model (CCM, [3]) proposed that the psychobiological response to competition would be better understood within a general stress model. According to the CCM, psychological and cognitive factors (e.g., perceived self-efficacy, motivation, or competitiveness) are core variables in explaining response to competition and its result. Prior to the commencement of the competition, an assessment of the contest takes place, considering both distal factors (e.g., prior history or personality) and proximal factors (e.g., motivation), culminating in a situational appraisal. This cognitive appraisal stems from an equilibrium between the demands of the situation and the perceived resources to manage it. When an individual perceives the situation as significant and controllable, the likelihood of a challenge appraisal rises, accompanied by the adoption of active coping strategies characterized by an increase in positive mood and activation of the sympathetic nervous system [4]. On the other hand, an imbalance between demands and resources leads to a threatening appraisal, related to passive coping strategies [3]. This different perception could influence the probability of winning or losing.

According to the CCM model, distal factors, such as personality, influence and modulate the appraisal of the specific competition. The literature underscores the significance of traits like competitiveness and anxiety. Competitiveness is characterized by the enjoyment of interpersonal competition and the desire to win and surpass others [5]. This personality trait is considered a predictor of achievement approach–performance motivation [6]. Elevated competitiveness has the potential to induce a challenge state when resource appraisals are also high, thereby facilitating victory. Moreover, individuals with high levels of competitiveness may perceive a competitive situation more positively, prompting them to set more ambitious objectives, which are correlated with enhanced performance [7]. In the competitive context, the data indicate that there is a positive relationship between competitiveness and performance [6,8]. Anxiety has also received attention from researchers in the field of competition but for different reasons. Specifically, trait anxiety is considered a reflection of the individual’s tendency to perceive the situation as threatening, experience higher state anxiety, and have lower performance during competitive tasks. In sports competitions, a relationship has been observed between trait anxiety and self-efficacy, confidence [9], and state anxiety [10], indicating that people with low anxiety showed lower levels of state anxiety than those with moderate or high anxiety, as well as higher positive self-efficacy levels. These results could reflect the impact of trait anxiety on the situational appraisal, increasing the probability of a threat assessment. In addition, trait anxiety has been negatively related to performance [11], suggesting that it could have an indirect influence on performance and outcome. In this sense, higher levels of pre-competitive anxiety have been found in losers than in winners [12], and anxiety has been established as a discriminant factor that can classify winners and losers [13,14]. In sum, personality traits can modulate the way individuals perceive and cope with competition, so they might have an indirect impact on performance and the outcome of the competition. Competitiveness and trait anxiety have been related to challenge and threat states, as well as to performance [8,10].

Challenge and threat states are also a consequence of proximal factors such as motivation or self-efficacy. In this scenario, motivation to compete is a necessary condition to elicit the psychobiological response related to competitive behavior and outcome. However, depending on the situation perception, the importance, and the task engagement, motivation can be related to challenge or threat states [15]. Approach goals would lead individuals to perceive an upcoming competition as a challenge [16,17], thus facilitating performance and victory. Self-efficacy perception before competition acts as a moderator of the effort and resources invested to win the competition [18]. It has been identified as an important component of the resource appraisal related to coping and performance in competitive situations [18,19]. Individuals possessing high levels of self-efficacy would perceive the situation as a challenge, predisposing them to confront it actively and enhance their likelihood of victory. Conversely, individuals with low self-efficacy would exhibit the opposite response [3]. Self-efficacy has been positively related to effort and performance [20] in learning contexts. Specifically in competition, higher levels of self-efficacy have been reported in winners [21].

Moreover, competition also entails significant physiological demands. A physiological challenge response is typically marked by heightened cardiac activity, while in contrast, a threat response is characterized by more modest increases in cardiac activity [22]. The challenge state would lead individuals to reach a higher performance level [23,24] and, consequently, increase the probability of winning. The reverse pattern would manifest in the threat state. Heart rate (HR) serves as a comprehensive index of mixed sympathetic–parasympathetic activity widely employed in competition research, representing a significant variable in the assessment of challenge and threat responses. It has been posited that an increase in HR serves as an indicator of task engagement, a characteristic shared between both challenge and threat states [22], although it could be higher in challenge states [16], positively related to effort [25] and, in competitive contexts, with an outcome based on skills, as in our study, winners have shown higher HR than losers [21,26]. Hence, in a competitive setting, both winners and losers may experience increases in heart rate (HR), though these increments are expected to be more pronounced in winners.

In summary, researchers across diverse fields, including academia, sports, and the workplace, share an interest in investigating individual differences in coping with competition and performance to anticipate outcomes. Nevertheless, as the outcome of the competition is not solely contingent on individual performance, we deem it crucial to ascertain the specific influence of individual characteristics that forecast success or failure.

Hence, the overarching objective of our research was to scrutinize the impact of specific psychobiological factors on the likelihood of winning or losing in a competitive setting. More precisely, our aim was to elucidate the predictive efficacy of distal factors (trait competitiveness and trait anxiety) and proximal factors (self-efficacy, motivation, and HR reactivity) in achieving victory within a face-to-face laboratory competition conducted with a cohort of young, healthy university participants. To address this inquiry, we conducted a laboratory competition, capturing both psychological and cardiovascular variables. The selection of these variables was grounded in their significance within competitive contexts and their established associations with challenge and threat states, as well as performance outcomes. Our hypothesis is that winners will manifest elevated levels of competitiveness, self-efficacy, motivation, and HR reactivity, coupled with reduced anxiety levels.

## 2. Materials and Methods

### 2.1. Participants

To guarantee the consistency of the results, three independent studies were carried out with different samples, using a within-subjects random assignment design. These studies were approved by the Ethics Research Committee of the University of Valencia (protocol code H1393232860606) in accordance with the ethical standards of the Declaration of Helsinki (1964). All the participants received verbal and written information about the main characteristics of the study and signed an informed consent form before beginning the experiment.

To qualify for participation in these studies, individuals had to meet specific inclusion and exclusion criteria. Inclusion criteria encompassed being healthy young university students. On the contrary, exclusion criteria included smoking (more than 5 cigarettes per day), regular drug use, engaging in intensive exercise training (more than 10 h per week), having cardiovascular, neurological, or psychiatric diseases, and using medication that could potentially impact psychophysiological responses.

#### 2.1.1. Sample 1

Seventy-eight students (30 men and 48 women) participated in this study. Participants were selected based on a pre-selection questionnaire. Individuals who met any of the following criteria were excluded: presence of a cardiovascular or psychiatric disorder, drug use, physical activity of more than 10 h per week, and the use of medication that could alter cardiac activity. Once they had been selected, participants were contacted by telephone and asked to maintain their normal intake and sleep patterns, avoid alcohol consumption, and refrain from performing extreme physical activities during the 24 h prior to the experiment. After the experimental phase, one participant was eliminated due to irregularities in the acquisition of the electrophysiological record. Therefore, the final sample in the first study consisted of 77 participants (37 winners and 40 losers) who were 21.8 ± 3.32 years old (mean ± SD) and had a body mass index (BMI) of 22.6 ± 3.32.

#### 2.1.2. Sample 2

In the second phase of this study, 36 male university students from different faculties at Miguel Hernández University in Elche were recruited. These participants met the same inclusion criteria and participated in an identical experimental procedure as in study 1. After the competition, 18 winners and 18 losers were obtained. The mean age was 21.2 ± 1.75, and BMI was 24.9 ± 3.57.

#### 2.1.3. Sample 3

In the third phase, 40 female university students from different faculties at the University of Valencia were recruited. These participants met the same inclusion criteria and participated in the same experimental procedure as in study 1. After the competition, 20 winners and 20 losers were obtained. The mean age was 22.6 ± 2.65, and BMI was 21.68 ± 2.

### 2.2. Measures

#### 2.2.1. Competitiveness

Interpersonal competitiveness (IC) was evaluated with the Competitiveness Questionnaire [27]. This scale refers to the desire to win, and it has a high internal consistency index (α =0.76). Participants expressed their degree of agreement on a Likert scale (from 1 to 5) with items such as: I have always wanted to be better than others.

#### 2.2.2. Anxiety

Trait anxiety (TA) was evaluated with the State Trait Anxiety Inventory [28]. The trait scale refers to the tendency to perceive situations as more threatening. The internal consistency of this scale was α =0.90. Participants expressed their degree of agreement with the items on a Likert scale (from 1 to 5).

#### 2.2.3. Motivation

Based on previous studies [26], we employed the following item: How important is it for you to win this competition? To evaluate the degree of motivation (M) oriented toward winning the competition. Participants answered this item on a Likert scale from 1 to 100. 

#### 2.2.4. Self-Efficacy

Self-efficacy (SE) was measured before the competitive task with the following item: How capable are you of successfully performing the competition task? Prior to the study, we conducted an inter-rater reliability test to find out if this item represented the self-efficacy construct described by Bandura [29]. The result was a kappa index = 1. The self-efficacy item was answered by the participants on a Likert scale from 1 to 100. 

#### 2.2.5. Heart Rate Reactivity

HR activity was recorded using a Polar RS800CX monitor (CIC polar, Kempele, Finland). This instrument is composed of a chest strap that detects cardiac activity and a clock that records cardiac information. Polar RE800CX devices have demonstrated exceptional reliability and validity for recording cardiovascular activity through changes in body position [30]. HR was calculated in two 5 min periods: one measurement at baseline and one during the competition. HR reactivity (HRR) was calculated with a simple subtraction of competition levels from baseline levels.

### 2.3. Competitive Task

The task employed in this study was a modification of the squares and letters test [31]. This is a paper-and-pencil test that consists of a sheet with different matrixes (4 * 4) containing 16 letters. The task consisted of finding the letter that is repeated in each matrix as quickly as possible. In order to involve the participants in a competition, the task was modified by introducing some elements: (a) The participants sat face-to-face at opposite ends of the same table; (b) The researcher informed the participants that they would compete for an economic prize (5 EUR); and (c) The task was divided into 5 rounds (2.5 min each), after which the participants received feedback about their performance. The winner of the competition was determined by the real performance on the task: number of matrixes completed correctly minus the incorrect matrixes.

### 2.4. Procedure

The day before the experiment, participants were asked to maintain their regular habits and avoid drinking alcohol and strenuous physical activity. Two hours beforehand, they were instructed not to drink stimulants or smoke. All participants performed the same procedure.

**Figure 1:**
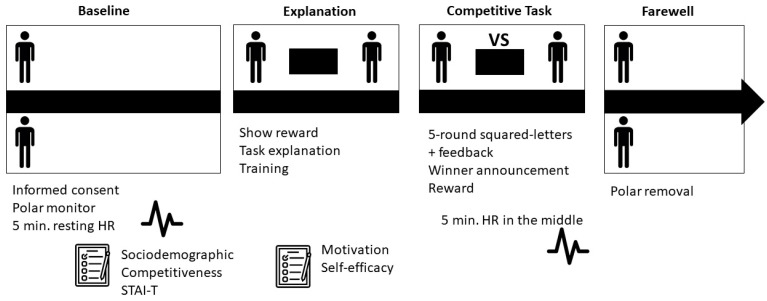
Study protocol.

The experimental sessions were carried out between 16:00 and 20:00 in order to control circadian rhythms in cardiovascular activity. Two people of the same sex participated in each experimental session. When participants arrived at the laboratory, they were each seated in a separate individual room where they received information about the experimental procedure and signed an informed consent form. Subsequently, the experimenter instructed participants to adjust the Polar monitor for the HR registration, and s/he asked the participants to remain seated and relaxed for the next 10 min. The last five minutes of this period were used as the basal measure of HR. Next, the researcher provided a series of trait questionnaires (competitiveness and trait anxiety) and sociodemographic information and instructed the participants to complete them. Once both participants had finished the questionnaires, they were moved to a common room where they sat face-to-face at the same table. At this time, the researcher communicated to the participants that they were going to compete for an economic prize; s/he explained the task and gave participants a small sample of the task so that they could become familiar with it. After this training and before the competition, the participants answered some questions about self-efficacy and motivation. Afterward, they carried out the competition, which lasted about 20 min (5 rounds of 2.5 min plus the correction time and feedback between the rounds). The central 5 min of the competition period was used to obtain the competition measurement of HR. At the end of the competition, the experimenter announced the winner and rewarded him/her with the prize. After announcing the winner, participants were instructed to return to their individual rooms and remove the Polar monitor (Figure 1).

### 2.5. Analyses and Data Reduction

Although it was not a main objective of this research, the first step in the analysis consisted of analyzing the effect of sex on the measured variables in order to control this effect in subsequent analyses. Through successive tests, we verified that there were differences in the means of heart rate reactivity (HRR) (t_[37.10]_ = 2.591; *p* < 0.05, |d|= 0.662), self-efficacy (SE) (t_[72]_ = 2.161; *p* < 0.05, |d| = 0.813) and motivation (M) (t_[72]_ = 2.061; *p* < 0.05, |d| = 0.553). Only HRR was a heteroscedastic variable (Z_[72]_ = 8.765; *p* < 0.05). Nevertheless, in all three cases, the variables were distributed in a similar way, as we found after applying the Kolmogorov–Smirnov test for two independent samples.

These results led us to conclude that, despite the sex differences in the means, they did not affect the distribution of the variables, and, therefore, it was possible to minimize the effect of gender by transforming the direct scores into a scale of normalized or Z scores. The variables in which the sex had shown a significant effect were typified according to the men’s and women’s means and standard deviations. The other variables were typified according to the global means and standard deviations. When a case was detected that exceeded three standard deviations, it was considered an outlier and eliminated from the analysis. Hence, the final sample consisted of 66 valid cases. Finally, the typical scores were transformed into T scores, which verifies that their means have an approximate average of 50 and a standard deviation of 10. Table 1 shows the preprocessed and typified descriptive statistics of the variables included in the analyses.

Subsequently, we verified that the variables fulfilled the necessary statistical assumptions to carry out the discriminant analysis (DA) using sample 1. We included in the model trait anxiety (TA), self-efficacy (SE), interpersonal competitiveness (IC), motivation (M), and heart rate reactivity (HRR) as independent variables, to check the capacity of the model to predict outcome (win vs. lose). Multivariate normality was assumed when it was verified that the independent variables were all distributed according to the normal distribution; likewise, the equality of the variances–covariances matrixes was verified (M de Box = 23.715, *p* = 0.116). Once the DA had been performed, a confirmatory analysis was conducted with the second and third samples in order to show the validity of the predictions of the first sample. Given that the variables fulfilled the parametric assumptions, we checked their effect on the dependent variable by comparing their averages using the *t*-test for independent samples. Afterward, it was tested collinearity by means of the correlation matrix. Finally, discriminant analysis was performed using the block introduction method.

All the analyses were performed with the statistical package SPSS.

## 3. Results

### 3.1. Preliminary Results

In Table 2, we observed that IC and HRR were the only variables with significantly different averages between the groups and a medium-magnitude effect size. Although the SE, M, and TA measures were not significant, we decided to keep them in the discriminant model due to their importance in the literature presented in the introduction.

The analysis of the determinant of the correlation matrix between the predictors showed the existence of certain collinearity between the variables (det|A| = 0.681), and this was later confirmed in the matrix of correlations between the variables (Table 2). We found that M, SE, and IC were positively correlated. Despite this problem of collinearity among the predictors, we ruled out the possibility of combining them linearly to predict the outcome of the competition, in order to avoid a significant loss of variability as much as possible.

### 3.2. Discriminant Analyses

After verifying that our variables met the necessary conditions for the application of the DA, we constructed the model using the block introduction method. We obtained a single canonical discriminant function with a λ = 0.123 and a canonical correlation coefficient of 0.331. This coefficient, together with the value of the Lambda de Wilks obtained (λ = 0.890), led us to consider that our model has modest explanatory power (Table 2 also shows the coefficients of the discriminant function). In the model, we confirm that HRR, IC, and M are the best predictors of winning/losing the competition.

Despite the modest explanatory power of the model, we found that its predictive power was substantial. The set of variables considered made it possible to correctly classify 68.8% (n = 53) of the cases introduced. The model’s predictive capacity of the winning vs. loser profiles is similar, without detecting a different performance in the classification based on the profile of the participant. The rate of correct classifications for the losing profile was 73% (n = 27), whereas for the winning profile, it was 65% (n = 26).

We also checked that our model’s predictions were not an independent variable of the subject’s outcome. Using an independence test, we verified that the two variables were related $\chi_{\left[1\right]}^{2}$ = 11.133; *p* < 0.001 with a moderate magnitude (φ|66| = 0.380; *p* < 0.001).

### 3.3. Confirmatory Analyses

Given that our model had low explanatory power but an interesting predictive capacity, we wanted to find out whether this predictive capacity could be extended to another sample, or if it was a spurious effect due to the overfitting of the model. To determine this, we verified that the model constructed in the first study was able to predict the result of the same competition in another set of participants different from those used to estimate the coefficients of the discriminant function. That is, we set out to test its generalization capacity in another set of participants.

First, we examined whether the distribution of the scores in the independent variables of the model for the generalization sample was similar to the distribution observed in the first sample in which the coefficients of the discriminant function had been estimated. Table 3 shows that the averages and standard deviations of these variables in the generalization sample were similar to the descriptive statistics obtained previously in the first sample (see Table 2 and Table 3). This similarity is verified by non-significant Levene contrasts for the variances of the groups and the t values for each pair of averages (Table 3 column 2).

Next, to determine whether each subject belongs to the winner vs. loser group, each of his/her scores typified on the independent variables was weighted by the coefficients of the discriminant function (Table 3). The cut-off point that made it possible to assign subjects to the winning or losing group was obtained from the calculation of the average distance between the centroids of the winning group and the losing group. The rate of correct classifications in validation sample 1 (only men) was 60.71%, and this percentage was slightly lower than expected. The rate of correct classifications for the losing profile was 57.14%, whereas for the winning profile, it was 71.43%. In the same way, we repeated the procedure with a second validation sample (only women). The rate of correct classifications in validation sample 2 was 57.14%, with 56.52% for the losing profile and 58.33% for the winning profile.

## 4. Discussion

The aim of this study was to scrutinize the explanatory impact of psychophysiological variables on competition outcomes. The findings underscored the substantial predictive capability of the variable set in classifying participants as winners or losers based on these parameters. Notably, competitiveness and heart rate reactivity emerged as the most robust predictors of competition outcomes, followed by motivation and self-efficacy, with trait anxiety exhibiting the least influence. Consequently, our model accurately forecasted competition outcomes in 68.8% of cases, demonstrating consistent predictive efficacy for both winners (65%) and losers (73%). Furthermore, the results indicated the replicability of this model across diverse samples of both men and women. Although there was a slight decrease in predictive power in the validation samples, the outcome remained statistically significant.

Proximal situational factors (motivation, self-efficacy, and HR reactivity) turned out to be significant predictors of outcome. That is, winners would be characterized by high scores on motivation, self-efficacy, and HR reactivity. These results are consistent with what was expected and with the challenge appraisal hypothesis described in the CCM [3] and also with the theory of challenge and threat states in athletes [16], which proposed that, in competitive situations, high self-efficacy and motivation oriented toward approach goals would lead to appraising the competition as a challenge, characterized by an increase in HR and positive mood, thus increasing the chances of success. The opposite would lead to a threat state associated with a lower cardiac response and negative mood, increasing the probability of defeat.

In addition, these results are in line with previous literature that showed higher self-efficacy in winners and a relationship between self-efficacy, approach achievement goals, and competition performance [18,32]. This might occur because confidence about performing the competitive task successfully would lead to facing the competition as a challenge, as long as the situation is important enough for the individual, and he/she is more focused on approach goals than on avoidance goals [16]. On the other hand, and in agreement with previous studies [21,26], our data reveal that high HR reactivity during competition would be a characteristic of winners. A high HR response could be a consequence of the challenge state produced by the situational appraisal. Thus, in a challenge state, a parasympathetic withdrawal/sympathetic activation, characterized by HR increases, would prepare the organism to actively cope with the competitive situation [3], thus increasing the chance of victory.

As demonstrated earlier, the outcome of a competition is influenced not only by situational factors but also by significant distal factors. In our investigation, we have substantiated the predictive potency of interpersonal competitiveness and anxiety, with competitiveness emerging as the most explicative distal variable. As anticipated, winners exhibited elevated scores in interpersonal competitiveness and lower scores in anxiety. It is plausible that this heightened competitiveness fosters a greater inclination toward enjoyment of competitive situations. Drawing on the motivation model of achievement, individuals with heightened interpersonal competitiveness may be motivated to assert their superiority over others [33]. Consequently, they may engage in task-approaching mechanisms, predisposing them to enhance the likelihood of winning.

In the context of competition, individual traits and the participants’ contextual appraisals play crucial roles in predicting outcomes. This study’s notable contribution lies in its proposition and substantiation of a psychophysiological profile distinguishing winners from losers. This complements prior research that has indicated a winner profile marked by elevated confidence levels and diminished anxiety [13,14]. The main implications are that training these variables could increase the probability of reaching victory. It is worth noting that all the variables introduced in the model are, in some way, sensitive to training. In the case of competitiveness, it has been pointed out that receiving feedback on one’s performance during training would improve performance, in addition to increasing motivation and competitiveness [34]. Therefore, providing adequate feedback during training would be a key factor in increasing the probability of winning. Training trait anxiety directly may pose challenges, but interventions aimed at bolstering positive self-perception, enhancing individual capacities, and mitigating anxiety states have the potential to yield long-term reductions in trait anxiety. Additionally, efforts to enhance self-efficacy and motivation can be fruitful. In this context, training programs targeting the reinforcement of beliefs in personal control, outcome expectations, motivation, and effort have been suggested as effective avenues for improving self-efficacy [35,36]. Regarding baseline heart rate (HR), exploring training methodologies such as biofeedback or cardiac coherence could prove beneficial. Previous studies have demonstrated the efficacy of these techniques in enhancing an individual’s control over their HR response in competitive and stressful scenarios [37,38,39]. This enhanced control holds the potential to foster improved emotional and cognitive self-regulation, subsequently influencing performance outcomes.

This research, while insightful, is not without its limitations. The sample size warrants consideration, as a larger cohort could bolster the robustness of the results. Similarly, the constrained sizes of the validation samples imply that the misclassification of a single case could exert a notable influence on predictive power. Furthermore, it is worth noting that the analyzed variables exhibited a degree of correlation, suggesting the potential for positive transfers between characteristics through synergistic effects with targeted training. For future studies, broadening the scope to include additional situational variables like mood, heart rate variability (HRV), or total peripheral vascular resistance could enhance the applicability of the findings. Lastly, acknowledging the controlled laboratory setting, it is advisable to validate the model in real-world competitive scenarios for a more comprehensive assessment of its effectiveness.

## 5. Conclusions

This study demonstrates that various proximal and distal factors associated with competition have predictive capabilities regarding the outcome. Competitiveness, anxiety, self-efficacy, motivation, and heart rate reactivity emerge as effective predictors of success or failure in a controlled laboratory competition. In summary, our findings unveil a notable accuracy in classification rates, suggesting potential implications and applicability in diverse contexts and with different participant samples.

## Figures and Tables

**Table 1:** Descriptive statistics before the typification.

	Sample 1			Sample 2 (Men)	Sample 3 (Women)	Normality		
	Total (n = 66)	Women (n = 33)	Men (n = 33)	Total (n = 28)	Total (n = 35)	Sample 1	Sample 2	Sample 3
TA	20.879 (9.967)	21.049 (10.419)	20.6 (9.381)	19.143 (9.01)	19.225 (7.454)	0.621	0.569	0.697
SE	69.24 (16.107)	66.823 (16.947)	73.2 (14.059)	69.643 (15.271)	71.875 (14.662)	1.753 *	0.951	1.112
IC	22.742 (5.824)	23.08 (5.958)	22.537 (5.806)	22.857 (4.616)	22.450 (5.657)	0.405	0.534	0.447
M	50.773 (27.835)	54.878 (26.751)	44.04 (28.804)	57.857 (25.727)	60.750 (21.884)	1.088	0.671	0.425
HRR	8.328 (6.058)	10.152 (7.432)	7.214 (4.81)	6.88 (8.132)	4.793 (3.414)	1.083	0.538	0.138

* *p* < 0.05. TA (trait anxiety), SE (self-efficacy), IC (interpersonal competitiveness), M (motivation), HRR (heart rate reactivity).

**Table 2:** Statistics descriptive of the predictors for each group of the dependent variable, correlations between the independent variables, and coefficients of the discriminant function.

	Descriptives (T Scores)					Correlation Matrix					Coefficients of the Discriminant Function	
	Total	Loser	Winner	t	$\left|d\right|$	TA	SE	IC	M	HRR	Matrix of Structure	Standardized
TA	49.795 (10.068)	49.402 (11.544)	50.187 (8.503)	0.175	0.077	1					0.112	−0.063
SE	50.039 (9.815)	49.203 (10.161)	50.876 (9.538)	1.154	0.170	−0.062	1				0.245	0.071
IC	50.234 (9.574)	47.932 (10.175)	52.537 (8.47)	2.233 *	0.492	0.054	0.231 *	1			0.711	0.771
M	49.804 (10.201)	48.853 (11.329)	50.755 (9.009)	0.970	0.186	0.209	0.284 *	0.421 **	1		0.269	−0.185
HRR	49.715 (9.13)	47.598 (9.737)	51.832 (8.079)	2 *	0.473	0.167	0.158	0.064	0.224	1	0.684	−0.718

* p<0.05; ** p<0.01. Winner’ s centroid = 0.346; Loser’ s centroid = −0.346. TA (trait anxiety), SE (self-efficacy), IC (interpersonal competitiveness), M (motivation), HRR (heart rate reactivity).

**Table 3:** Descriptive statistics for the validation samples and contrast of means with the parameters of the estimation sample.

	Validation Sample 1		Validation Sample 2	
	Mean (sd)	t +	Mean (sd)	t †
TA	48.225 (9.326)	0.687	48.294 (7.154)	−0.875
SE	46.617 (11.127)	1.197	52.981 (8.652)	1.495
IC	54.014 (9.0644)	−1.574	48.943 (9.494)	−1.202
M	44.645 (8.899)	2.117 *	52.195 (8.181)	1.139
HRR	48.809 (7.542)	0.506	42.789 (4.593)	−4.793 **

* p<0.05; ** p<0.01. + The average is contrasted for each variable in the group of men in the sample for the estimation of the parameters of the discriminant function and among the averages in the validation sample. † The average is contrasted for each variable in the group of women in the sample for the estimation of the parameters of the discriminant function and among the averages in the validation sample. TA (trait anxiety), SE (self-efficacy), IC (interpersonal competitiveness), M (motivation), HRR (heart rate reactivity).

## Data Availability

Data will be available upon request from the corresponding author.
